# Factors influencing clinicians' willingness to use an AI-based clinical decision support system

**DOI:** 10.3389/fdgth.2022.920662

**Published:** 2022-08-16

**Authors:** Avishek Choudhury

**Affiliations:** Industrial and Management Systems Engineering, West Virginia University, Morgantown, WV, United States

**Keywords:** artificial intelligence, UTAUT, clinical decision support system, risk, expectations

## Abstract

**Background:**

Given the opportunities created by artificial intelligence (AI) based decision support systems in healthcare, the vital question is whether clinicians are willing to use this technology as an integral part of clinical workflow.

**Purpose:**

This study leverages validated questions to formulate an online survey and consequently explore cognitive human factors influencing clinicians' intention to use an AI-based Blood Utilization Calculator (BUC), an AI system embedded in the electronic health record that delivers data-driven personalized recommendations for the number of packed red blood cells to transfuse for a given patient.

**Method:**

A purposeful sampling strategy was used to exclusively include BUC users who are clinicians in a university hospital in Wisconsin. We recruited 119 BUC users who completed the entire survey. We leveraged structural equation modeling to capture the direct and indirect effects of “AI Perception” and “Expectancy” on clinicians' Intention to use the technology when mediated by “Perceived Risk”.

**Results:**

The findings indicate a significant negative relationship concerning the direct impact of AI's perception on BUC Risk (ß = −0.23, *p* < 0.001). Similarly, Expectancy had a significant negative effect on Risk (ß = −0.49, *p* < 0.001). We also noted a significant negative impact of Risk on the Intent to use BUC (ß = −0.34, *p* < 0.001). Regarding the indirect effect of Expectancy on the Intent to Use BUC, the findings show a significant positive impact mediated by Risk (ß = 0.17, *p* = 0.004). The study noted a significant positive and indirect effect of AI Perception on the Intent to Use BUC when mediated by risk (ß = 0.08, *p* = 0.027). Overall, this study demonstrated the influences of expectancy, perceived risk, and perception of AI on clinicians' intent to use BUC (an AI system). AI developers need to emphasize the benefits of AI technology, ensure ease of use (effort expectancy), clarify the system's potential (performance expectancy), and minimize the risk perceptions by improving the overall design.

**Conclusion:**

Identifying the factors that determine clinicians' intent to use AI-based decision support systems can help improve technology adoption and use in the healthcare domain. Enhanced and safe adoption of AI can uplift the overall care process and help standardize clinical decisions and procedures. An improved AI adoption in healthcare will help clinicians share their everyday clinical workload and make critical decisions.

## Introduction

The growth of Artificial Intelligence (AI) has been gradually shifting the healthcare paradigms over the last decade. According to most technical experts in biomedical informatics, AI will revolutionize many medical fields in the near future (Bohr and Memarzadeh, 2020; Kohane, Drazen, & Campion, 2012). In subspecialties such as radiology, AI technologies have outperformed clinicians (1), and AI is getting more efficient at performing clinical tasks beyond diagnosis and early detection. In 2020, the US Food and Drug Administration approved an AI software that provides real-time guidance to medical professionals and thus enabling them to perform cardiac ultrasound imaging without specialized training (2). Another study proposed a deep learning model that could precisely predict patients' needs in the critical care department (3). Given the research trend and investment in AI research, AI technology, in the future, is likely to become an integral part of the healthcare ecosystem, where clinicians and AI should work in a systematic collaboration.

There is much evidence indicating the positive impact of AI on healthcare. However, whether clinicians (the end-user) will adopt or use the technology is an ongoing concern. Not just the fears of being replaced by AI technologies, several other factors such as myths, reliability, resilience, the inexplicability of AI, and unfamiliarity with the technology might determine clinicians' intent to use AI. Many recent studies have been dedicated to addressing the technical challenges of AI, mainly developing the explainability and reliability of the technology (4). Still, not much work has been invested in understanding how these technologies are perceived by clinicians, mainly existing AI users, and *do they want to use them*? (5) Medical professionals often consider the potential of AI to be limited (6), and their perceptions can impact their intent to use or adopt AI in medicine. Therefore, it is important to understand the human factors that influence clinicians' intent to use AI; otherwise, AI would remain underused, keeping the healthcare industry benefiting from the technology.

## Theory and related work

**Figure 1:**
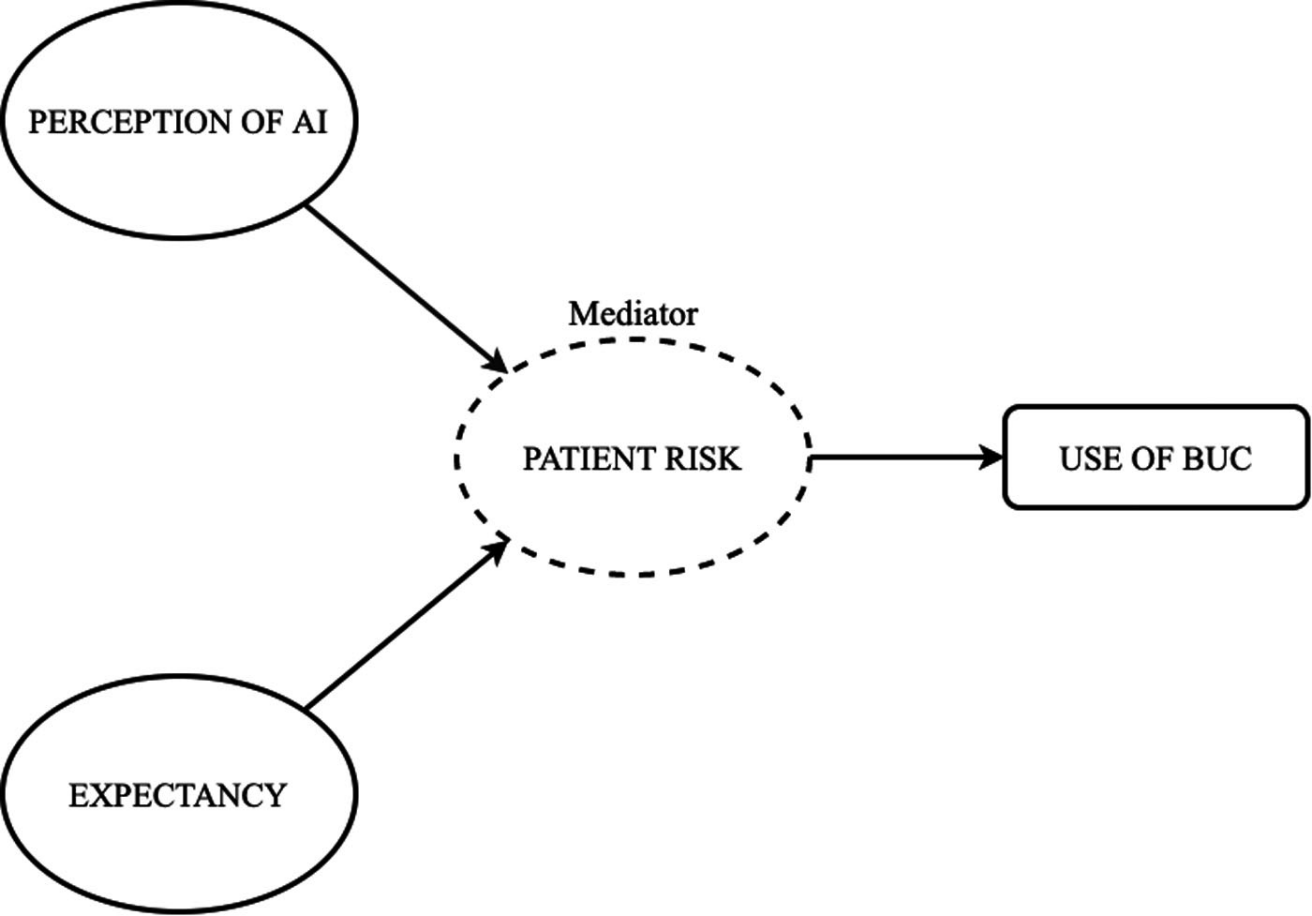
Conceptual framework.

Patients and medical professionals are the most important and potential users of AI-based applications. They often express concerns about implementing AI-based tools in the care services (7, 8). In our study, the concerned AI (the Blood Utilization Calculator-BUC) is a clinician-facing clinical decision support system directly impacting patient outcomes. Therefore, clinicians are concerned about the impact it may have on them. Clinicians' perceptions regarding the BUC may significantly steer the adoption and use of the technology. Researchers need to explore the current challenges of AI use and adoption from a human factor standpoint. One way to do so is by analyzing the antecedents of risk beliefs and expectancies associated with using AI-based devices (BUC) from the clinician's perspective (Figure 1). There is a lack of evidence showing risk beliefs and individuals' withdrawal from using AI clinical devices (9). But studies examining the impact of expectancy and general perception of AI on intent to use BUC, mainly when mediated by perceived risk, from consumers' (AI users) perspectives, have not been investigated.

Nevertheless, several studies analyzing healthcare AI systems from human factors standpoint leverages various acceptance models not limited to the technology acceptance model (TAM), the unified theory of acceptance and use of technology (UTAUT) (10), and the value-based adoption model, stating that consumer behaviors (clinicians) towards new technology (BUC) rely on their perceptions of that technology (AI) in general (Chung and Koo, 2015). TAM captures the mediating role of perceived ease of use and perceived usefulness between system characteristics and its use. Studies have leveraged TAM to explain users' behavior toward technology. Studies using the UTAUT primarily explored the effects of four core factors (performance expectancy, effort expectancy, social influence, and facilitating conditions) on user acceptance of a technology and usage behavior. However, existing studies have not captured the impact of value perceptions (benefit and risk beliefs) or the general perception of clinicians associated with BUC, which may influence their perception of risk and intention to use the technology (11). Thus, in our study, we hypothesized that the general *perception of AI would influence clinicians' perceived risk of BUC – Hypothesis 1*.

Expectancy theory states that individuals have choices (for clinicians, it is whether to develop their reasoning or accept BUC recommendations), and their decisions are driven by the way they perceive that a particular action (intent to use BUC) will lead to the best outcome (minimal risk to themselves and their patients) (12). Similarly, the Theory of Planned Behavior (TPB) and the Theory of Reasoned Action (TRA) capture the importance of users' beliefs regarding the outcome, normative expectations, possible hindrances, and the ability to control the process, in determining their behavior (13). We bring effort expectancy and performance expectancy together as a second-order latent construct for expectancy. We also included perceived risks together in a theoretical synthesis. These concepts interact with *expectancy* and *perception of AI* in ways that help shape BUC users' behavioral intention and hypothesized that BUC expectancy *would negatively influence its perceived risk – Hypothesis 2*.

The perception of risk regarding any system can decrease the utility attached to the technology (14). Being a complex and developing technology, AI-based devices (BUC) are not yet an integral component of the healthcare system or medical training, and the ambiguity about the safety and risks that an AI can impose (15) on patients are still decisive factors that facilitate users' intention to use the technology. Similarly, clinicians will typically support and use AI technologies if they believe that they will augment healthcare delivery and patient safety outcomes without undermining their values. In other words, the perceived benefits of AI technologies will motivate clinicians to use them in the future (8).

Well-established theories like the Task Technology-Fit (TTF) state that a user will only adopt a given technology when it fits their need and consecutively improves their performance (16). Many studies have leveraged TTF to explain the technology adoption (17). That being said, if clinicians perceived AI as a technology that would augment their clinical practice and meet their requirements, the likelihood of AI adoption in healthcare would increase. Several studies have showcased the promising potential of AI applications within the healthcare system, creating a positive perception of AI in society (18–22). The perception of risks and safety can influence a clinician's intention to use AI systems (8). External influences and uncertainty associated with AI use can create biases among clinicians, which may encourage or keep them from using AI technologies in the future. In this study, we use the variable “risk” as a mediating construct and hypothesized that *perceived risk would mediate the effect of expectancy on the use of BUC – Hypothesis 3 and will also mediate the effect of perception of AI on the use of BUC – Hypothesis 4*.

## Problem statement

Healthcare AI can be a promising medium to expedite effective care and fulfill the global shortage of medical resources. However, we still lack sufficient empirical evidence and human factors perspective capturing clinicians' perceptions of medical AI. As an emerging technology, effective and safe integration of AI into the existing healthcare system depends on numerous technological challenges and whether the medical professionals are willing to use it. As acknowledged by Davenport and Kalakota, “*the greatest challenge to AI in these healthcare domains is not whether the technologies will be capable enough to be useful, but rather ensuring their adoption in daily clinical practice”* (6).

The AI technology studied in this research is a Blood Utilization Calculator (BUC), a module of an electronic decision support program known as the Digital Intern (iVMD). This AI system is a proprietary computer-based algorithm that retrieves patient information from the electronic medical record and delivers data-driven personalized recommendations for the number of packed red blood cells to transfuse for a given patient (23). The AI is developed to optimize blood transfusion and protect patients from infectious agents and alloimmunization, which may occur due to excessive blood transfusion (23, 24). According to prior studies, BUC was more consistent than clinicians (24); however, clinicians only accepted about 49% of BUC recommendations (25), deterring its use in the hospital. Therefore, to understand the factors influencing clinicians' intention to use BUC or accept BUC recommendations, we implemented human factors approach and explored clinicians' (BUC users) perception of BUC. We specifically aimed to understand how the perception of AI, risk, and expectancy influences clinicians' intention to use BUC. To explore the intended effects, we analyze the conceptual model as illustrated in Figure 1 and test the following four hypotheses:

**H1.** The general perception of AI will have a negative effect on the perceived risk of BUC.
**H2.** The expectancy of BUC will have a negative effect on its perceived risk.
**H3.** Perceived risk of BUC will mediate the effect of expectancy on the intent to use BUC.
**H4.** Perceived risk of BUC will mediate the effect of perception of AI on the intent to use BUC.

## Material and method

The study was conducted at a university hospital in Wisconsin, US. Before the study began, all participants (clinicians) were briefed on the in-depth study intent. All methods were carried out following relevant guidelines and regulations. The study obtained ethical approval from the University of Wisconsin, Madison, USA (IRB ID 2020-1110). It was determined to meet the criteria for exempting human subjects' research per the category(ies) defined under 45 CFR 46.

### Data collection / recruitment

The study targeted medical professionals who use BUC. We distributed a mass email to the list servers of clinicians who worked in the hospital. The email described the purpose of the study, a description of BUC, and a link to the online survey. Interested clinicians participated with consent. The survey was distributed between February 2021 and July 2021. We used RedCap to collect survey responses. The survey contained a screening question asking whether they have ever used the BUC system (with an explanation and picture of BUC). Only BUC users were asked to complete the survey. We discarded incomplete and duplicate responses. Each participant completing the survey was given a $20 gift card. No participant identifiers were obtained during the study.

### Participants

We received 273 individual responses in total. One hundred nineteen healthcare professionals were BUC users (said Yes to the screening question) and completed the entire survey. The remaining 154 were not BUC users and did not complete the rest of the questions in the survey. About 73.9% of respondents were Caucasian Americans, and 81.5% were physician residents (they were the primary users of the BUC), followed by 11.8% attending physicians and 6.7% nurses. We also note that 68.9% used BUC for up to two years, and 29.4% used BUC for three to five years. The majority of the participants were female (53.8%). About 90% of the participants were aged between 25 and 35 yrs.

### Instrumentation

The study adapted validated questions from the modified, extended unified theory of acceptance and use of technology (UTAUT-2) model (26, 27), as shown in Supplementary Appendix A. The UTAUT 2 is a theoretical framework derived from the Theory of Planned Behavior and the Technology Acceptance Model (28). According to this framework, an individual's intention to use a technology depends on factors such as the performance expectancy (i.e., the degree to which the technology is perceived to be useful) and effort expectancy (i.e., the degree to which using the technology is perceived to be easy to use) (29). Our survey questions were intended to measure expectancy, risk, and intention of using BUC. We define expectancy as a second-order latent construct consisting of effort expectancy and performance expectancy. Perception of risk indicated the likelihood that patient health will deteriorate if exposed to an event [decision based on wrong BUC recommendation] (30). We also included a question to measure clinicians' overall perception of AI, mainly how clinicians think an AI [for instance, BUC] will improve patient outcomes. All questions were modified from their original form to fit the context of this research focusing on AI (BUC) and medical professionals.

### Statistical analyses

We calculated descriptive statistics of the survey responses and Pearson correlations to show the related variables. We then conducted a discriminant validity test to ensure the square roots of AVEs do not exceed the correlation coefficients of paired latent constructs. We also calculated the Variance Inflation Factor (VIF) and tolerance values for the predictor variables and checked for multicollinearity. Since self-reported surveys are often prone to biases, we used Harman's single factor test to test for common method bias. All analyses about correlation, discriminant validity, biases, and multicollinearity tests were performed in SPSS Version 27.

The confirmatory factor analysis (CFA) was performed using a structural equations approach to the survey measures to analyze the psychometric properties of “effort expectancy,” “performance expectancy,” “perception of AI,” and “perceived risk.” To ensure the fit of “expectancy” as a single construct consisting of “effort and performance expectancy,” we conducted second-order CFA. The fit and reliability of the constructs to the data were determined as acceptable as indicated by Composite reliability (CR), average variance extracted (AVE), Guttman's lambda 6, and coefficient omega (for second-order CFA of expectancy). The SEM encompasses multiple regression analysis that allows a simultaneous estimate of the direct and indirect causal relationships between variables; therefore, it is preferred in cognitive modeling and behavior analysis (Lowry and Gaskin, 2014). The final structural model was evaluated using indicators such as the Goodness of Fit Index (GFI), Comparative Fit Index (CFI), and Tucker Lewis index (TLI). We also conducted a mediation modeling using structural equation modeling (SEM), controlling for “*age,” “race,” “clinical experience,”* and “*experience with BUC*,” to capture the predictive relationships between “expectancy” and “use of BUC,” mediated by “trust.” The control variables were included as covariates in the model predicting “perceived risk” and “intent to use BUC.” No significant effects of age, race, and experiences were found and therefore were dropped to improve model fit.

All confirmatory factor analysis and structural equation modeling were performed using “lavaan” and “psych” packages in RStudio version 1.4.1717. The reproducible code for the confirmatory factor analysis and SEM is available in the zenodo repository (31).

## Results

Clinicians agreed that AI systems could improve patient outcomes (mean 3.97, max 5) and disagreed that the use of BUC can put them or their patients at risk (mean 1.95 and 1.83, respectively). Clinicians also perceived BUC as an easy-to-use AI system (mean 3.76); they agreed that learning how to use BUC and becoming skillful at it was easy (mean 3.81 and 3.82, respectively). Most of the clinicians neither agreed nor disagreed with the question asking if the BUC increased their chances of achieving/fulfilling important clinical tasks (mean 3.33). However, most of them agreed that BUC improved their pace (mean 3.36) and effectiveness at blood transfusion (mean 3.64).

### Common bias method and multicollinearity

We also conducted Harman's one-factor test to check for common method bias (32). All factors together explained 51.28% of the total variance (greater than 50%), and we acknowledge common method bias as a limitation of our study (33, 34). We calculated the Variance Inflation Factor (VIF) and tolerance values for the predictor variables to check for multicollinearity. All VIFs were below the cutoff value of 5 and ranged between 1.4 and 1.6. The tolerances were also higher than the recommended threshold of 0.1 and ranged between 0.6 and 0.9 (35). Thus, no multicollinearity was observed in this research.

### Confirmatory factor analysis and discriminant validity

As shown in Supplementary Appendix B, the confirmatory factor analysis assesses the adequacy of latent constructs involved in this study. The proposed measurement model fit the data adequately well (chi-square = 23.56, CFI = 0.99, TLI = 0.98, RMSEA = 0.05, and *p*-value = 0.13). All factor loadings were significant and greater than 0.70, indicating acceptable loadings. The obtained measures meet the requirements of (36) and (37), showing evidence of convergent validity. All reported AVE values were greater than 0.5, satisfying the minimum requirement (38).

We also tested for discriminant validity and leveraged the Heterotrait-Monotrait Ratio (HTMT) Technique. The HTMT technique indicates the similarities between different latent variables. HTMT of less than 0.85 indicates a reliable discriminant validity (39). In our study, HTMT was noted to be 0.48, ensuring discriminant validity.

### The structural equation model

**Figure 2:**
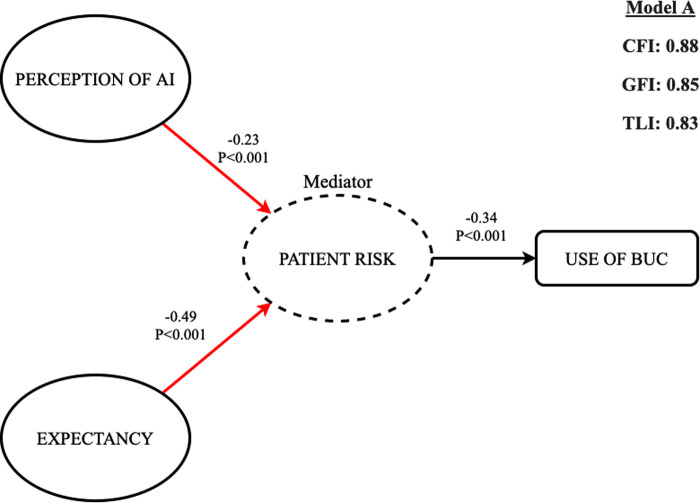
Schematic illustration of the structural equation modeling [CFI = 0.88, GFI = 0.85, TLI = 0.83].

The final structural model fit evaluated using indicators such as the GFI, CFI, and TLI indicated a marginal fit (>0.80 and <0.90). Figure 2 illustrates the standardized path coefficients of the structural model under investigation. The structural model was assessed by examining path coefficients. We also calculated the significance of each path. The results of hypotheses testing (direct and indirect effects) are summarized in Table 1. The findings support hypothesis 1 by showing a significant negative relationship (ß = −0.23, *p* < 0.001). Similarly, expectancy significantly affected risk (ß = −0.49, *p* < 0.001) and supports hypothesis 2. We also noted a significant negative impact of Risk on the Intent to use BUC (ß = −0.34, *p* < 0.001). Regarding the indirect effect of expectancy on the intent to use BUC, the findings support hypothesis 3 by showing a significant positive impact on the intent to use BUC when mediated by risk (ß = 0.17, *p* = 0.004). Hypothesis 4, which posits that risk will mediate the relationship between perception of AI and intent to use BUC, was also supported. We noted a significant positive and indirect effect of perception of AI on the intent to use BUC when mediated by risk (ß = 0.08, *p* = 0.027)

**Table 1:** Effects of expectancy, perceived risk, and trust on the intention of using the BUC.

Effects	Standardized estimate (ß)	Standard error	z-value	*p*-value
Model A				
Expectancy → Risk	−0.49	0.15	−4.14	<0.001
Perception of AI → Risk	−0.23	0.07	−2.65	<0.001
Risk → Use of BUC	−0.34	0.15	−3.75	<0.001
Expectancy → *Risk *→ Use of BUC	0.17	0.12	2.87	0.004
Perception of AI → *Risk *→ Use of BUC	0.08	0.05	2.21	0.027

## Discussion

Given the increasing availability of AI systems in healthcare, such as BUC systems, the essential question is how perceived risk is influenced and whether clinicians are willing to accept and use this technology as an integral part of their routine clinical practices. This is the first study to leverage the expectancy theory and UTAUT 2 framework to explore the significant roles played by the general perception of AI, expectancy, and how these factors influence the perceived risk of this system and, eventually, clinicians' intention to use BUC.

In our study, expectancy (effort and performance expectancy) ranged from neutral to moderately high among most clinicians. Our findings showed a significant negative impact of expectancy on perceived risk, i.e., as the expectancy of BUC increased, clinicians perceived the technology as a low-risk AI system where perceived risk can be defined as a perception of conviction that a clinician would sustain a loss when they seek an outcome (40). Our finding that poor expectancy can worsen risk perception is consistent with the literature. Although no other studies have evaluated BUC in particular, the interactions between expectancy, risk and intent to use technologies have been established in the literature. A 2019 study demonstrated a negative effect of expectancy on the mobile technology field's perceived risk (26). Another study in 2021 measured the impact of perceived risk and effort expectancy on the adoption of AI (41). According to our analysis, the extent to which clinicians believed that AI technologies (not BUC in particular) would improve patient outcomes was inversely related to how they perceived BUC as a high-risk technology. We also noted that the clinicians' intent to use the technology decreased with an increased perception of risk regarding BUC. In other words, the clinicians' perception of AI had a significant positive indirect influence on the clinician's intent to use BUC (a particular AI system). Although no prior studies have captured the exact interaction for direct comparisons, the notion that injunctive social influence (referring to what clinicians think about AI technologies in general) could influence technology use was established in the Theory of Reasoned Action (42).

Most clinicians in our study perceived BUC as a low-risk technology. We also note that perception of risk is a significant mediator. In other words, perceived risk was a significant influencer of the intent to use BUC and mediated the effects of expectancy and perception of AI. In the same line, a 2014 study stated that risk factors are crucial in mobile services, and the higher the risk of using new technology, the lower the willingness to use (43). A 2011 study showed that perceived risk significantly influences users' attitudes towards technology acceptance (44). In 2018, a study argued that perceived risk could significantly reduce intent to use a technology (45). To our knowledge, most related studies have examined perceived risk as an external factor influencing the external variables of the UTAUT model (46), and no prior studies have examined perceived risk as a mediating factor between expectancy, perception of AI, and intent to use AI.

Few studies have used the human factors approach and inspected various AI-based systems across different domains (11); there is still a lack of understanding of how clinicians' perception of AI-based decision support systems and their expectancies influence their risk perception and intent to use the technology. Previous studies have primarily investigated non-users' intention to use AI using technology acceptance theories, including TAM and UTAUT (47, 48). Our study captures the perception of AI users. This is important because the perception of AI might change with time and job sensitivity (when patient health is at stake), as the AI-generated recommendations influence patient health outcomes. Different people define AI differently and have different expectations of AI.

Measuring AI perception of individuals who never used AI cannot reflect their experiences with the technology, but their biases and opinions formed by external factors such as news media or the experimental setup of a particular study. Most technology acceptance models were developed for the non-intelligent systems (49) and often oversee the effect of different human factors on the perception of risk. In the context of healthcare AI technologies, clinicians are very likely to prioritize the risk factor, i.e., whether an AI-based decision support system is safe for their patients and can deliver good quality recommendations. Thus, there is a need for AI developers to understand the potential factors that can influence users' perception of AI risk. Due to the specificity of the healthcare field, we proposed the perception of risk as a mediating factor. Our study also has practical implications. In this study, clinicians' positive perceptions toward BUC expectancy can lead to a lower perception of risk and, in turn, result in a higher intention to use BUC. Emphasizing the potential benefits such as (a) rapid calculation to determine the required units of blood needed for a transfusion, (b) accuracy of recommendation, (c) reliability of data analysis, and (d) consistency with the clinical requirements may increase clinicians' intention to use the technology.

Moreover, the concerns and challenges associated with BUC risk perception substantially negatively impact the intention to use BUC. If clinicians cannot reduce risk concerns, they may prefer traditional human-human interaction and calculate their own decisions over AI. We also found that clinicians' perception of AI, in general, determines their risk perception towards BUC in particular and consequently reduces their intention to use BUC. Therefore, when hospitals want to incorporate a specific AI system into their workflow, they should ensure that the potential users of the technology are aware of the system (BUC) and its functioning. Management should also ensure that clinicians are not influenced by unscientific news regarding AI (myths and hypes). Within healthcare, addressing the concerns contributing to risk beliefs about AI is a priority.

Future studies should focus on the ethical and regulatory considerations associated with AI technologies (50). Accountability can also be a significant influencing factor in the acceptance of AI. In the context of our study, clinicians (the stakeholder) should be informed about the accountability and regulations in cooperation with healthcare institutions. The concerned management should develop a handbook clearly stating how AI-based BUC was designed, how it abides by the ethical principles (such as fairness and health equity), and the potential risks. The BUC should be more transparent to the clinicians from a human factor standpoint. Timely external validation of the BUC can also help clinicians understand the risks and benefits of BUC. Basic training of the clinicians regarding the functioning of the BUC (without violating the proprietary norms) should be encouraged by the management. In line with the literature (51, 52), we also suggest that future studies should measure clinicians' intent to use AI technology over a more extended period as their attitudes and perception of AI may change with the change of experience. We believe that when clinicians are more aware of the BUC or AI decision support systems in general, external influences (perception of available AI technologies), impractical expectations from the technology, or wrong perceptions of risks will not hinder their willingness to use them BUC. The intention of whether to use BUC (AI) will only be a function of its effectiveness and impact on patient outcomes.

There are three limitations of this study that must be acknowledged. **(a)** This study focuses on a particular AI solution (BUC) used by clinicians at a single hospital. It also consists of two single-item measures (Perception of AI and Intent to use BUC). Since the predictive validity of single-item measures, when used in conjunction with multi-item scales, depends on particular conditions, our findings cannot be generalized across other AI technologies; **(b)** Although sample size sufficiency for the estimation of structural equation modeling (SEM), suggests at least 100 observations, the robustness of estimates should be interpreted keeping in mind the limited sample size of this study (53); **(c)** We also identified the presence of common method bias in the survey responses.

## Conclusion

The rapid advances in AI technologies will inevitably shape the healthcare system, health communications, and clinical workflow. The maximum benefits of AI technologies in healthcare can be realized when there is a safe and systematic implementation of AI devices. Thus far, several research has documented the power and potential of AI technologies within healthcare institutions. However, the integration of advanced systems such as AI in healthcare mandates a sound understanding of the technology and the human factors responsible for hindering technology acceptance among clinicians. In an inpatient setting, clinicians are one of the most critical stakeholders of AI technologies. Our model suggests that a clinician's perception of risk is a crucial factor. Due to the nature of healthcare services, the implementation of AI should be performed with specific considerations.

In summary, this study demonstrated that significant and indirect influences of expectancy and perception of AI on the use of the BUC were mediated *via* perceived risk. AI developers need to emphasize the benefits of AI technology, ensure ease of use (effort expectancy), clarify the system's potential (performance expectancy), and minimize the risk perceptions by improving the overall design. Future research and management policies should encourage the participatory involvement of clinicians (all stakeholders) and ensure defined accountability and responsibility of healthcare professionals while using AI technology, as these measures can potentially minimize risk perception and improve their intent to use the technology. Identifying the factors that determine clinicians' intent to use AI-based decision support systems can help improve technology adoption and use in the healthcare domain. Enhanced and safe adoption of AI can uplift the overall care process and help standardize clinical decisions and procedures. An improved AI adoption in healthcare will help clinicians share their everyday clinical workload and make critical decisions. Not only in blood transfusion-related tasks, AI acceptance and safe integration will also improve overall care quality and facilitate timely intervention. If appropriately designed and used, AI can also augment home care and self-diagnosis for certain ailments. However, further research is needed to confirm the effectiveness when primarily used by patients during home care or self-care.

## Data Availability

The raw data supporting the conclusions of this article will be made available by the authors, without undue reservation.
