# Supplementary material for: Factors influencing clinicians' willingness to use an AI-based clinical decision support system
Source: Front Digit Health. 2022 Aug 16;4:920662. doi: 10.3389/fdgth.2022.920662 (PMC9628998; doi:10.3389/fdgth.2022.920662)
Supplement: Supplementary file 1 [file Datasheet1.pdf]

Appendix A

Survey questions

Factors	Questions	Scale
**Perception of AI**	I think artificial intelligence systems have the potential to improve patient outcomes	5 Likert scale (1 = Strongly Disagree to 5 = Strongly Agree)
**Perception Risk**	"Using the BUC puts me at an overall greater risk."	5 Likert scale (1 = Strongly Disagree to 5 = Strongly Agree)
	"Using the BUC exposes my patients at overall greater risk."	
**Expectancy**	**Effort expectancy**	
	Learning how to use the BUC is easy for me	5 Likert scale (1 = Strongly Disagree to 5 = Strongly Agree)
	I find the BUC easy to use	
	It is easy for me to become skillful at using the BUC	
	**Performance expectancy**	
	"Using BUC has increased my chances of achieving things that are important to me."	5 Likert scale (1 = Strongly Disagree to 5 = Strongly Agree)
	“Using BUC allows me to perform tasks more quickly."	
	"Using BUC has increased my effectiveness in blood utilization calculation/transfusion."	
**Use of BUC**	"If it were up to you, to what extent would you use BUC?"	5 Likert scale (1 = Almost Never to 5 = Very Often)
