# Supplementary material for: Factors influencing clinicians' willingness to use an AI-based clinical decision support system
Source: Front Digit Health. 2022 Aug 16;4:920662. doi: 10.3389/fdgth.2022.920662 (PMC9628998; doi:10.3389/fdgth.2022.920662)
Supplement: Supplementary file 2 [file Datasheet2.pdf]

Appendix B

Confirmatory factor analysis model assessment

Factor structure (from CFA)		Reliability			
**First Order CFA**					
	**item**	**Loadings**	**AVE**	**G6**	**CR**
**Effort Expectancy**	Learning how to use the BUC is easy for me	0.89	0.79	0.89	0.91
	I find the BUC easy to use	0.97			
	It is easy for me to become skillful at using the BUC	0.80			
**Performance Expectancy**	Using BUC has increased my chances of achieving things that are important to me	0.71	0.64	0.971	0.89
	Using BUC allows me to perform tasks more quickly	0.87			
	Using BUC has increased my effectiveness in blood utilization calculation/transfusion	0.75			
**Perceived Risk**	Using the BUC puts me at an overall greater risk	0.87	0.91	0.971	0.90
	Using the BUC exposes my patients at overall greater risk	0.97			
**Second-Order CFA**					
	**item**	**Loadings**	**Omega**	**G6**	**CR**
**Expectancy**	Performance Expectancy	0.91	0.80	0.91	0.90
	Effort Expectancy	0.83			
